# A Comparative Study of Clinical Presentation and Risk Factors for Adverse Outcome in Patients Hospitalised with Acute Respiratory Disease Due to MERS Coronavirus or Other Causes

**DOI:** 10.1371/journal.pone.0165978

**Published:** 2016-11-03

**Authors:** Musa A. Garbati, Shamsudeen F. Fagbo, Vicky J. Fang, Leila Skakni, Mercy Joseph, Tariq A. Wani, Benjamin J. Cowling, Malik Peiris, Ahmed Hakawi

**Affiliations:** 1 King Fahad Medical City, Riyadh, Saudi Arabia; 2 School of Public Health, The University of Hong Kong, Hong Kong SAR; Chinese University of Hong Kong, HONG KONG

## Abstract

Middle East Respiratory syndrome (MERS) first emerged in Saudi Arabia in 2012 and remains a global health concern. The objective of this study was to compare the clinical features and risk factors for adverse outcome in patients with RT-PCR confirmed MERS and in those with acute respiratory disease who were MERS-CoV negative, presenting to the King Fahad Medical City (KFMC) in Riyadh between October 2012 and May 2014. The demographics, clinical and laboratory characteristics and clinical outcomes of patients with RT-PCR confirmed MERS-CoV infection was compared with those testing negative MERS-CoV PCR. Health care workers (HCW) with MERS were compared with MERS patients who were not health care workers. One hundred and fifty nine patients were eligible for inclusion. Forty eight tested positive for MERS CoV, 44 (92%) being hospital acquired infections and 23 were HCW. There were 111 MERS-CoV negative patients with acute respiratory illnesses included in this study as “negative controls”. Patient with confirmed MERS-CoV infection were not clinically distinguishable from those with negative MERS-CoV RT-PCR results although diarrhoea was commoner in MERS patients. A high level of suspicion in initiating laboratory tests for MERS-CoV is therefore indicated. Variables associated with adverse outcome were older age and diabetes as a co-morbid illness. Interestingly, co-morbid illnesses other than diabetes were not significantly associated with poor outcome. Health care workers with MERS had a markedly better clinical outcome compared to non HCW MERS patients.

## Introduction

MERS coronavirus (MERS-CoV) was initially identified in a 60-year-old male with severe progressive fatal pneumonia in Jeddah, Saudi Arabia in June 2012 [1]. As of July 24th, 2016, 1,782 patients have been confirmed with MERS-CoV infection globally with 634 fatalities [2]. MERS still remains a zoonotic infection with dromedary camels suspected to be the source of infection [3,4]. However, transmission between humans can occur within family or health care settings, some of these leading to hundreds of infections, as occurred in Jeddah, Riyadh and the Republic of Korea [5]. Outbreaks within health care facilities have been associated with low clinical suspicion and delayed recognition, leading to unsuspected secondary transmission. Other factors predisposing to such transmission events include over-crowded emergency rooms, lack of effective triage at first contact with the health care system, deficiencies in routine infection control practices and mild unrecognised infections, especially among health care workers (HCW) resulting in outbreaks [5,6,7,8].

Earlier studies have attempted to describe the clinical features of MERS as well as compare it with SARS [9, 10, 11]. However, there is still a paucity of data comparing the clinical presentation of MERS with those of patients who were suspected of MERS but tested MERS-CoV negative. Information on whether or not there are clinical, demographic or epidemiological characteristics that distinguish MERS from other causes of acute respiratory illness (ARI) is important for clinicians providing care in MERS-CoV endemic countries such as Saudi Arabia, or when caring for travellers returning from MERS-endemic areas. In one study, 17 patients with MERS were compared with 82 concurrently investigated patients who were RT-PCR negative for MERS [12]. Furthermore, while there have been reports on the clinical presentation of MERS in HCW [13], we could not identify previous studies that compared the clinical features of MERS-CoV infected health care workers with those who are non-health care workers.

To address these gaps in knowledge, in this study we sought to analyse the demographic characteristics, clinical data and outcomes of all patients meeting the case definition for MERS at a major hospital in Riyadh between 2012 and 2014 who were tested by RT-PCR for MERS-CoV. The clinical features and outcomes of those virologically confirmed to be MERS-CoV positive and negative were compared. In addition, we compared MERS-CoV positive HCW with MERS-CoV positive non-HCWs. The molecular epidemiology of a subset of these patients has been previously reported [7].

## Methods

### Clinical Setting

The study was conducted at King Fahad Medical Centre (KFMC), a 1200-bed tertiary care hospital in Riyadh which comprises of four hospitals (Main, Women’s Specialized, Childrens’ and Rehabilitation); the main hospital houses four affiliated centres (National Neurosciences Institute, Comprehensive Cancer, Diabetes and Endocrine) in addition to the Medical and Surgical Specialty departments within the same campus. The intensive care unit (ICU) is located in the main hospital. The medical city serves as a tertiary referral centre for patients from throughout the Kingdom.

### Patients and Specimens

Included in this study are patients who met the clinical case definition for MERS and were tested by RT-PCR for MERS-CoV RNA between October 2012 and May 2014. A confirmed case of MERS was defined by the Ministry of Health, Saudi Arabia as a febrile patient with ARI who tested positive for MERS-CoV infection. Patients with community- or nosocomially-acquired MERS as well as HCW diagnosed with MERS at KFMC were included. Patients meeting the clinical case definition during the same time frame who were MERS-CoV negative served as controls. Nasopharyngeal swabs or lower respiratory tract specimens (tracheal aspirates, bronchoalveolar lavage) were collected from patients meeting the clinical case definition of MERS and tested for presence of MERS-CoV RNA.

### Laboratory Diagnosis

From October 2012 to April 2014, samples were tested for MERS-CoV at the MOH laboratories. At the end of April 2014 when in-house MERS-CoV testing capability was developed, all samples were tested at KFMC. RT-PCR diagnosis for MERS-CoV was based on WHO recommendations, testing the upstream and using Orf1a for confirmation [14].

### Demographic and Clinical Data

Patient demographic and clinical data were retrieved from electronic health records and via retrospective chart review.

### Statistical Analysis

We compared various characteristics among MERS-CoV positive HCW, MERS-CoV positive non-HCW, and MERS-CoV negative, cases, by Fisher’s exact test for categorical factors or by ANOVA for continuous factors. We looked at factors associated with poor outcome (admission to ICU or death) among MERS patients, and compared the factors between patients with poor versus favourable outcome by Fisher’s exact test or ANOVA. In addition, we looked at the factors associated with death in MERS patients by fitting a logistic regression. We investigated time from hospital admission to death among subgroups, by fitting Kaplan-Meier curves, and compared subgroups by the log-rank test.

### Ethical Issues

The study was approved by the King Fahad Medical City Institutional Review board. Given that this was an outbreak situation they waived the requirement for individual informed consent.

## Results

The demographic characteristics of 159 eligible patients were reviewed. Forty eight of the patients were confirmed to be MERS-CoV RT-PCR positive while 111 were negative in MERS-CoV RT-PCR tests (Table 1). Of the 48 MERS patients, 44 were hospital acquired and 23 HCW. Health care workers with MERS were more likely to be expatriates than MERS-CoV infected non-HCW (p<0.01) or MERS-CoV negative (p<0.01) patients. However, there was no difference between non-HCW MERS-CoV positive and MERS-CoV negative patients in this regard. Of the 23 HCW with MERS, 21 were from the Philippines, one was from Malaysia and one from India. Although MERS-CoV positive patients were overall more likely to be female (68.8% vs 49.6%; p = 0.04), the non-HCW MERS-CoV positive patients were not significantly different in this regard from MERS-CoV negative patients (56.3% vs 49.6%; p = 0.28). MERS patients who were non-HCW were more likely to be older (median age 60 years) than non-MERS patients (median age 33 years) (p<0.01) and HCW MERS patients (median age 36 years)(p = 0.01). The non HCW MERS patients were all persons who had been attended the hospital emergency room or those who were admitted for other reasons and acquired infection within the hospital.

**Table 1:** Demographics of MERS positive HCW, MERS positive non-HCW and MERS negative cases.

	1.Positive HCW cases (N = 23)		2.Positive non-HCW cases (N = 25)		3.Negative cases (N = 111)		p-value (1 vs 2)	p-value (2 vs 3)	p-value (1+2 vs 3)
	n	(%)	n	(%)	n	(%)			
Age group, in years							<0.01	0.04	<0.01
0–20	0	(0%)	1	(4%)	34	(31%)			
21–30	3	(13%)	3	(12%)	13	(12%)			
31–40	12	(52%)	3	(12%)	16	(14%)			
41–50	4	(17%)	1	(4%)	9	(8%)			
51–60	3	(13%)	5	(20%)	14	(13%)			
>60	1	(4%)	12	(48%)	25	(23%)			
Median (range)	36	(24–74)	60	(12–77)	33	(0–91)	0.01	<0.01	0.02
Male	6	(26%)	9	(36%)	56	(50%)	0.67	0.28	0.04
Saudi	1	(4%)	24	(96%)	101	(91%)	<0.01	0.69	<0.01

A history of exposure to animals (camel, dog or poultry) was more common in the MERS-CoV positive patients overall, compared with MERS-CoV negative patients (p = 0.04) (Table 2). A history of exposure to ill persons in a health care setting in the two weeks prior to onset of illness was more frequent in non- HCW MERS-CoV positive (95%) than in MERS-CoV negative (62%) patients (p = 0.01).

**Table 2:** Comparison of potential exposure factors among the three patient groups.

Characteristics	1.Positive HCW cases (N = 23)		2.Positive non-HCW cases (N = 25)		3.Negative cases (N = 111)		p-value (1 vs 2)	p-value (2 vs 3)	p-value (1+2 vs 3)
	N	(%)	n	(%)	n	(%)			
Travel history	0	(0%)	1	(5%)	14	(15%)	0.48	0.30	0.04
Animal exposure history^#^	1	(4%)	2	(10%)	0	(0%)	0.59	0.04	0.04
Exposed to a health care setting*	23	(100%)	21	(95%)	58	(62%)	0.98	0.01	<0.01
Exposure duration							>0.99	0.04	0.01
0-4hr	6	(26%)	5	(23%)	45	(50%)			
> = 5hrs	17	(74%)	17	(77%)	45	(50%)			

*-two weeks prior to onset of illness

^#^ Animal exposure of HCW was to a dog and of non-HCW with MERS was to a camel and poultry

The presenting clinical features and co-morbidities of MERS-CoV positive (HCW and non-HCW) and MERS-CoV negative patients are shown in Table 3. Comparatively, MERS-CoV positive (HCW and non-HCW and negative patients had similar clinical presentations and co-morbidities, except for diarrhoea that was more frequent in the MERS-CoV positive cohort. The frequency of reporting diarrhoea in the MERS-CoV positive HCW, MERS-CoV positive non-HCW and MERS-CoV negative patient groups was 11/23 (50%), 5/25 (23%) and 15/111 (16%) respectively. A statistically significant difference was observed when the frequency of diarrhoea was compared between the MERS-CoV positive patients (either HCW or non-HCW) and MERS-CoV negative patients (p = 0.02). Within the MERS-CoV positive cohort, shortness of breath, tachycardia and underlying co-morbidities (such as cancer, diabetes, hypertension and heart disease) were significantly more frequent amongst non-HCWs than in HCWs.

**Table 3:** Comparison of clinical symptoms and co-morbidity status among the three patient groups.

	1. Positive HCW cases (N = 23)*		2. Positive non-HCW cases (N = 25)*		3. Negative cases (N = 111)*		p-value (1 vs 2)	p-value (2 vs 3)	p-value (1+2 vs 3)
	n	(%)	n	(%)	n	(%)			
Sign & Symptoms									
Fever	20	(87%)	24	(96%)	90	(81%)	0.54	0.13	0.15
Sore throat	7	(32%)	9	(45%)	18	(21%)	0.58	0.05	0.06
Cough	14	(64%)	20	(83%)	91	(86%)	0.24	>0.99	0.12
Dyspnoea	6	(26%)	19	(83%)	63	(64%)	<0.01	0.13	0.38
Hemoptysis	1	(4%)	1	(5%)	9	(10%)	>0.99	0.74	0.48
Wheezing	3	(13%)	6	(29%)	19	(20%)	0.37	0.60	>0.99
Tachycardia	1	(4%)	14	(61%)	40	(42%)	<0.01	0.17	0.37
Abdomen pain	7	(32%)	6	(27%)	19	(21%)	>0.99	0.70	0.35
Vomiting	5	(23%)	8	(33%)	25	(27%)	0.64	0.76	>0.99
**Diarrhea**	**11**	**(50%)**	**5**	**(23%)**	**15**	**(16%)**	**0.12**	**0.67**	**0.02**
Headache	9	(39%)	6	(27%)	17	(19%)	0.60	0.58	0.11
Runny nose	4	(18%)	6	(27%)	17	(19%)	0.72	0.55	0.75
Nausea	4	(17%)	5	(23%)	9	(10%)	0.94	0.20	0.17
Co-morbidity									
Cancer	0	(0%)	9	(38%)	15	(15%)	<0.01	0.03	0.71
Obesity	4	(17%)	8	(33%)	18	(18%)	0.36	0.18	0.42
Smoking	1	(4%)	1	(5%)	8	(9%)	>0.99	0.89	0.62
Diabetes	3	(13%)	14	(58%)	38	(37%)	<0.01	0.10	>0.99
HIV/other immune deficiency disease	0	(0%)	8	(35%)	16	(16%)	0.01	0.07	0.92
Steroid use	0	(0%)	1	(5%)	3	(3%)	0.98	>0.99	0.99
Hypertension	5	(22%)	15	(62%)	37	(37%)	0.01	0.04	0.61
Heart disease	0	(0%)	11	(46%)	31	(31%)	<0.01	0.24	0.47
Asthma	4	(17%)	3	(12%)	22	(22%)	0.95	0.46	0.45
CLD	0	(0%)	4	(17%)	7	(7%)	0.12	0.24	0.97
Haematological disorder	0	(0%)	4	(17%)	20	(20%)	0.12	>0.99	0.15
Pregnancy	0	(0%)	0	(0%)	4	(4%)	-	0.80	0.44

*Data on all clinical features were not available on all patients. % calculated based on denominator for which data was available.

The biochemical and haematologic parameters between the three groups were similar, except for higher white blood count (WBC) values in MERS-CoV negative patients and higher serum creatinine levels in MERS-CoV positive non-HCW compared with MERS negative patients (Table 4). There was no significant difference between the laboratory parameters of MERS-CoV positive HCW and non-HCW.

**Table 4:** Comparison of laboratory parameters among three patient groups.

	1.Positive HCW cases (N = 23)		2.Positive non-HCW cases (N = 25)		3.Negative cases (N = 111)		p-value (1 vs 2)	p-value (2 vs 3)	p-value (1+2 vs 3)
	μ	(95% CI)	μ	(95% CI)	μ	(95% CI)			
WBC	6.3	(4.7, 7.9)	7.5	(5.9, 9.1)	11.1	(9.6, 12.6)	0.30	0.03	<0.01
Lymphocyte	28.8	(23.9, 33.6)	21.6	(13.9, 29.3)	21.9	(19.1, 24.7)	0.16	0.93	0.29
Neutrophils	58.9	(50.5, 67.4)	69.6	(61.0, 78.3)	67.3	(63.6, 71.0)	0.09	0.60	0.49
Creatinine	70.0	(50.1, 90.0)	170.1	(78.3, 261.9)	103.1	(83.5, 122.7)	0.07	0.03	0.34
Creatine-k*	68.8	(-15.0, 152.6)	104.5	(-22.8, 231.8)	59.7	(28.5, 90.8)	0.78	0.33	0.37
Platelet count	254.1	(210.4, 297.8)	208.6	(156.6, 260.7)	264.3	(238.9, 289.8)	0.21	0.06	0.13
APTT	44.1	(39.9, 48.3)	44.0	(39.9, 48.1)	40.6	(38.4, 42.8)	0.97	0.17	0.10
LDH*	464.1	(354.4, 573.7)	516.1	(437.6, 594.7)	1368.7	(-249.0, 2986.5)	0.44	0.56	0.45

* Including some extreme large values.

Among the 48 patients with confirmed MERS, 47 had chest radiography available for review. Of these, 12 (25%) had normal chest radiographs at initial presentation, 21 (45%) had unilateral lung infiltrates and 14 (30%) had bilateral lung infiltrates. Seven (15%) of the total cohort never developed an infiltrate. Amongst those with normal initial radiographs, 3 (25%) and 2(17%) deteriorated and developed unilateral and bilateral infiltrates, respectively. Amongst patients with baseline unilateral infiltrates, 15 (71%) progressed and developed bilateral infiltrates; 4 (19%) had no changes. Two patients with bilateral infiltrates survived and had their lungs revert to normalcy.

Adverse clinical outcomes such as ICU admission or death were not significantly different between MERS-CoV positive (HCW and non-HCW considered together) and MERS-CoV negative patients. However, of the MERS-CoV positive HCW, only 4 of 23 (17%) required ICU admission and 1 of 23 (4.35%) died in contrast with 21 of 25 (84%) (p<0.001) and 16 of 25 (60%) (p<0.001) of the non-HCWs with MERS-CoV infection, respectively. Furthermore, non-HCW MERS-CoV positive patients were more likely to be admitted to ICU (21 of 25; 84%) than MERS-CoV negative patients (62 of 111 (56%) (p = 0.02); and were more likely to die (16 of 25; 60%) than MERS-CoV negative patients (25 of 111; 23%) (p<0.001) (Table 5).

**Table 5:** Comparison of poor outcome among three patient groups.

	1.Positive HCW cases (N = 23)		2.Positive non-HCW cases (N = 25)		3.Negative cases (N = 111)		p-value (1 vs 2)	p-value (2 vs 3)	p-value (1+2 vs 3)
	n	(%)	n	(%)	n	(%)			
Admission to ICU	4	(17%)	21	(84%)	62	(56%)	<0.01	0.02	0.79
Died	1	(4.3%)	16	(64%)	25	(23%)	<0.001	<0.001	0.12

Among the patients with MERS-CoV infection, a poor clinical outcome (ICU admission or death) was significantly associated with older age, presence of co-morbid conditions, lower lymphocyte count, higher neutrophil count, higher serum creatinine and higher serum LDH levels, by univariate analysis (Table 6). Diabetes was the co-morbid condition most strongly associated with adverse outcome (p = 0.0002). In multivariate regression analysis, factors that remained associated with a decreased risk of death were younger age and being a HCW (Table 7).

**Table 6:** Factors associated with poor outcome (admission to ICU or death) among 48 MERS patients.

	Poor outcome (N = 25)		Favorable outcome (N = 23)		p-value
	μ	(95% CI)	μ	(95% CI)	
Age (yr)	53.8	(46.7, 61.0)	39.5	(34.5, 44.5)	<0.01
Age (median, range)	60	(12, 77)	36	(22, 64)	<0.01
WBC (x10^9^/L)	7.5	(6.0, 9.0)	6.3	(4.6, 7.9)	0.29
Lymphocytes (x10^9^/L)	19.2	(14.0, 24.5)	31.7	(23.8, 39.6)	0.01
Neutrophils (%)	72.7	(66.1, 79.4)	55.1	(45.2, 64.9)	<0.01
Creatine (μmol/L)	176.3	(84.5, 268.2)	62.2	(55.5, 69.0)	0.04
Creatine-kinase (U/L)*	103.9	(-5.2, 213.0)	21.5	(20.5, 22.5)	0.65
Platelet count (x10^9^/L)	209.4	(162.5, 256.3)	253.1	(200.9, 305.3)	0.23
APTT (secs)	45.8	(41.6, 50.0)	40.3	(37.4, 43.3)	0.10
LDH (U/L)*	566.8	(487.3, 646.4)	369.8	(300.0, 439.6)	<0.01
	n	(%)	n	(%)	p-value
Age >60yr	11	(44%)	2	(9%)	0.02
No comorbid disease	3	(12%)	10	(43%)	<0.01
Diabetes with or without any other comorbid disease	15	(60%)	2	(9%)	
Any other comorbid disease without diabetes	7	(28%)	11	(48%)	

**Table 7:** Factors associated with fatal outcome in 48 MERS cases.

Characteristics	Risk of death	
	Adjusted OR	(95% CI)
Age group, in years		
0–45	1.00	
46–65	8.83	(1.26, 62.06)
65+	19.65	(1.39, 278.81)
Female	1.00	
Male	0.65	(0.08, 5.35)
Other occupation	1.00	
Heath care workers	0.12	(0.01, 0.91)
No comorbid disease	1.00	
With any comorbid disease	1.03	(0.06, 16.39)

**Fig 1:**
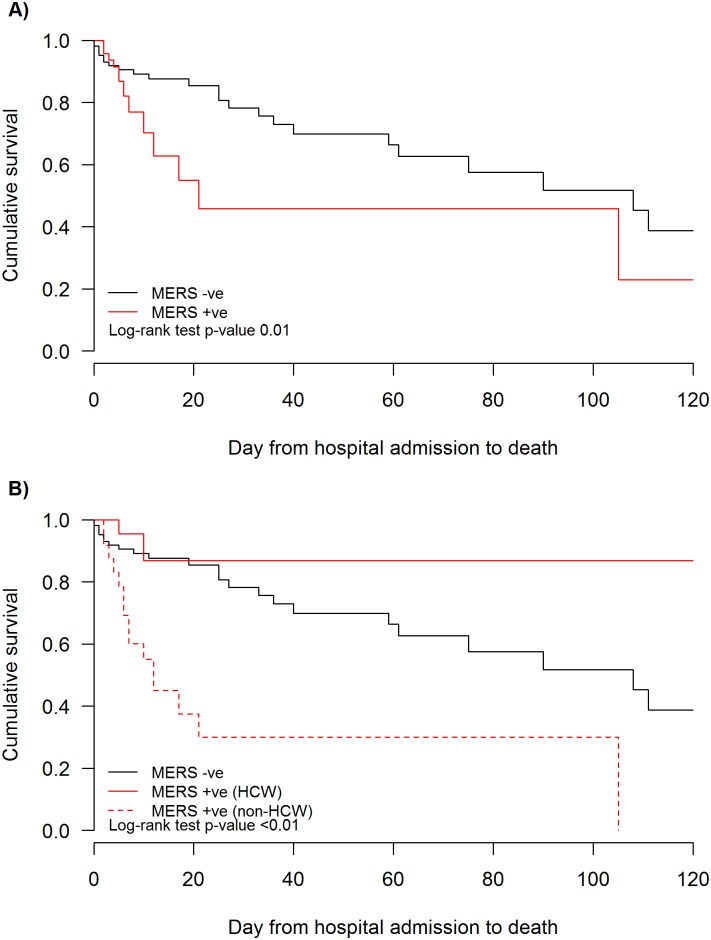
Time from hospital admission to death. A: Comparing patients with MERS-CoV infection (red line) and those negative for MERS-CoV (black line) (p = 0.01). B: Comparing health care workers with MERS CoV (red dotted line; group1), non-HCW with MERS (red solid line; group 2) and patients without MERS-CoV infection (black solid line; group 3). Log rank test group 1 vs. group 2 p = 0.003; group 2 vs group 3 p = 0.45; group 1 vs group 3 p<0.001.

**Fig 2:**
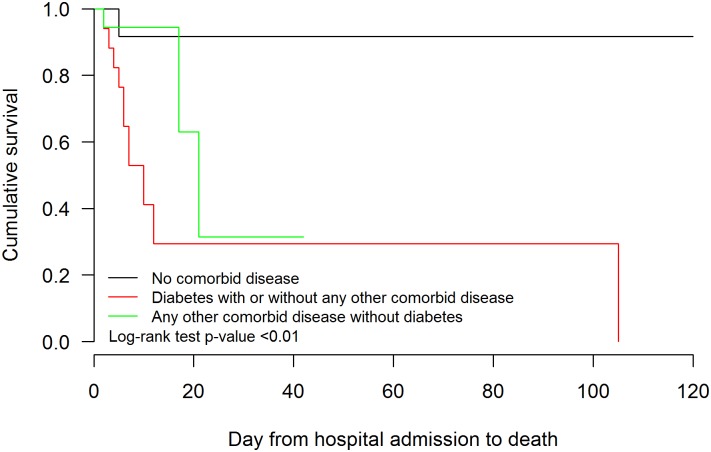
Time from hospital admission to death stratified by comorbid disease status among MERS positive cases. Survival analysis was carried out to ascertain impact of co-morbidities on survival in patients with MERS-CoV infection. Black line (group 1): No comorbid disease; Red line (group 2): Diabetes with or without other comorbidities; Green line (group 3): Any other comorbid disease without diabetes. Log rank test: group 1 vs group 2 p = 0.01; Group 1 vs. group 3 p = 0.54; group 2 vs. group 3 p = 0.01.

Using Kaplan-Meier analysis, survival was shown to be significantly different in patients with MERS-CoV infection (taken together) in comparison with MERS-CoV negative patients (Fig 1 upper panel). When MERS-CoV positive patients were stratified as HCW and non-HCWs, the former had better clinical outcome compared with non-HCW (p = 0.003) (Fig 1 lower panel). MERS positive non-HCW had worse outcome that MERS negative patients (p<0.001). Survival time was not significantly different between MERS-CoV positive HCW and MERS-CoV negative patients (p = 0.45). Survival analysis confirmed than MERS patients without co-morbidities had markedly better survival than those with diabetes (p = 0.01), but there was no significant difference compared with patients with co-morbidities other than diabetes (p = 0.54). Finally, MERS patients with diabetes had significantly worse survival that those with co-morbidities other than diabetes (p = 0.01) (Fig 2).

## Discussion

Clinical, demographic and outcome data from 159 patients investigated as suspected MERS were analysed; of these 48 were virologically confirmed as MERS-CoV infected. MERS patients who were not HCW were older than HCW and those who were MERS-CoV negative. The presenting clinical features of patients with MERS were indistinguishable from those who were investigated for MERS but found MERS-CoV negative, with the exception that diarrhoea was more common in patients with MERS. This appears to be primarily because 11 of 23 (48%) HCW with MERS reported diarrhoea significantly more often than patients who were MERS-CoV negative (15/94; 16%) (p = 0006). However, there was no difference in the frequency of diarrhoea in non-HCW MERS patients and non-MERS patients. Notably, diarrhoea was also a prominent feature of SARS with evidence of virus replication in intestinal epithelium [15]. It is important to note that 25% of patients had normal chest radiographs at initial presentation and 15% never developed an infiltrate. This was reminiscent of the radiological presentation of SARS where a proportion of patients also did not have an infiltrates on chest radiography at presentation but many of these did have detectable abnormalities on computerised tomography scans [15]. The observation that the clinical features of patients with MERS-CoV infection is indistinguishable from those who are hospitalised for other ARI emphasises the need for a low threshold of clinical suspicion for initiating MERS-CoV laboratory testing in patients with severe respiratory disease. Exposure to hospital environments in the two weeks prior to clinical onset was one significant risk factor for infection. However, 62% of patients who were not MERS-CoV positive also had similar exposure to health care facilities, possibly because many of them had other co-morbidities. Exposure to animals, though significantly more common reported in the non HCW patients with MERS (10%) than in those without MERS (0%), was not elicited in the majority of patients with MERS. Some of the MERS-CoV infections (both HCW and non-HCW) were known to have been acquired nosocomially [7] and thus would not be expected to have zoonotic exposure as a source of infection.

Patients with MERS who were non-HCW were more likely to > 50 years of age when compared to non-MERS patients (p = 0.0034) and MERS patients who were HCW (p = 0.0005). Patients with MERS who were non-HCW had significantly worse clinical outcomes than those who were MERS-CoV negative or HCW with MERS-CoV infection. This may be related, in part, to the fact that both HCW and non-MERS patient groups were younger and had fewer co-morbid conditions. In a previous report of MERS-CoV infection in HCW detected by active case finding of contacts, seven infected HCW were documented, two of whom were asymptomatic, while the other five had mild upper respiratory tract symptoms [13]. The age of the HCW with MERS-CoV infection ranged between 28–56 years with only one of them having underlying co-morbidity. Only 4 (17%) required ICU admission and one (4%) died. In other case series of MERS, significant proportions have been HCW, but the clinical features of the HCW and non HCW MERS patients have not been systematically compared [6,8,16]. It is clear that the overall clinical presentation and outcomes in HCW and non-HCW MERS is markedly different. When analysing clinical outcomes of MERS, it is important to distinguish between those in HCW and non-HCW, because they have markedly different clinical outcomes.

Taking all MERS patients together, the variables associated with poor clinical outcomes in univariate analysis were older age, presence of co-morbid illness, neutrophilia, lower lymphocyte counts, higher serum creatinine levels and higher serum LDH levels. On multivariate regression analysis, only occupation (HCW) and younger age were significantly associated with favourable clinical outcome. In survival analysis, MERS patients with diabetes (with or without other co-morbidites) had worse survival than MERS patients with other co-morbidities (other than diabetes) or those with no co-morbidities. Interestingly, patients with co-morbidities other than diabetes were not significantly different to those with no co-morbidities.

Limitations of the study include the possibility that some patients with MERS-CoV may have been missed because of false negative RT-PCR results and may be categorised as non-MERS-CoV patients. Convalescent sera were not available for testing from all patients under investigation. Secondly, the data collection was retrospective. Viral load data was not available for analysis. The MERS negative cases are likely to be heterogeneous in their diagnosis. Final discharge diagnosis of this control group is unfortunately not available.

In conclusion, MERS could not be reliably distinguished from patients who were MERS negative by clinical, demographic or epidemiological criteria; this highlights the need for a high index of suspicion, early isolation and early laboratory testing to identify infected patients and prevent nosocomial spread. An important observation from this study is that HCW with MERS had a markedly more favourable clinical outcome compared with MERS patients who were non-HCW, primarily because they were younger and had less co-morbidities. Clinical progression, presentation and outcomes are very different in these two groups and this has to be kept in mind and adjusted for when therapeutic trials for MERS are designed. Diabetes, rather than other co-morbidities had a significant adverse impact on survival.
